# Evolution of cooperation by the introduction of the probabilistic peer-punishment based on the difference of payoff

**DOI:** 10.1038/srep25413

**Published:** 2016-05-05

**Authors:** Tetsushi Ohdaira

**Affiliations:** 1 Institute of Information and Media, Aoyama Gakuin University, 4-4-25 Shibuya, Shibuya-ku, Tokyo 150-8366 Japan

## Abstract

There are two types of costly punishment, i.e. peer-punishment and pool-punishment. While peer-punishment applies direct face to face punishment, pool-punishment is based on multi-point, collective interaction among group members. Regarding those two types of costly punishment, peer-punishment is especially considered to have the flaws that it lowers the average payoff of all players as well as pool-punishment does, and facilitates antisocial behaviour like retaliation of a defector on a cooperator. Here, this study proposes the new peer-punishment that punishment to an opponent player works at high probability when an opponent one is uncooperative, and the difference of payoff between a player and an opponent one becomes large in order to prevent such antisocial behaviour. It is natural to think that players of high payoff do not expect to punish others of lower payoff because they do not have any complaints regarding their economic wealth. The author shows that the introduction of the proposed peer-punishment increases both the number of cooperative players and the average payoff of all players in various types of topology of connections between players.

Recent studies discuss whether costly punishment that a player paying some cost causes damage to another uncooperative player is effective in the increase and the maintenance of the number of cooperative players (the evolution of cooperation) in non-kin relations or not. Those discussions are based on either negative^1^,^2^,^3^,^4^,^5^ or positive^6^,^7^,^8^,^9^,^10^,^11^,^12^,^13^,^14^,^15^ point of view, and briefly explained as follows. Regarding negative discussions, in the prisoner’s dilemma game, Dreber *et al.*^2^ point out that the introduction of peer-punishment induces the decrease of the average payoff of all players as well as pool-punishment does. Peer-punishment, which is one of two types of costly punishment, means that a player directly punishes another uncooperative player by paying some cost. In the public goods game that is the multiplayer extension of the prisoner’s dilemma game, Rand and Nowak^5^ propose that peer-punishment is only a selfish tool to protect a player himself/herself because it facilitates antisocial behaviour like retaliation of a defector on a cooperator as a result of natural selection.

In contrast to the preceding argument, regarding positive discussions, Garcia and Traulsen^13^ show that Rand and Nowak^5^ discuss the quite limited case where a player abstains from collective action, and even if such antisocial behaviour is available to a player, a cooperator who only punishes a defector is able to prosper enough when those abstaining are isolated. Perc and Szolnoki^14^ propose adaptive punishment that a player is able to change his/her degree to punish another player in relation to the degree of success of cooperation. The adaptive punishment facilitates the reciprocity based on spatial connections between players (simply noted as connections below), and as a result enhances cooperation. Nakamaru and Dieckmann^15^ investigate the evolution of a social reaction norm, and find that (1) evolution towards enhanced cooperation and an ever more demanding punishment reaction norm mutually reinforce each other; (2) that mechanism works best when punishment is strict, i.e. ambiguities in defining defectors are small; (3) when the strictness of punishment is able to adapt jointly with the threshold and severity of punishment, evolution favours the strict-and-severe punishment that naturally evolves and leads to much enhanced cooperation when cooperation without punishment is weak and neither cooperation nor punishment are too costly; and (4) such evolutionary dynamics, through which defectors gradually and steadily evolve into cooperators who punish defectors, enable the bootstrapping of cooperation and punishment.

As for peer-punishment in the public goods game, Helbing *et al.*^16^ show that the consideration of punishment allows us to understand the establishment and spreading of cooperators who punish defectors. Szolnoki *et al.*^17^ study the impact of pool-punishment in the spatial public goods game with cooperators, defectors, and pool-punishers as the three competing strategies. Helbing *et al.*^16^,^18^,^19^ particularly show that the efficiency of pool-punishment in maintaining socially advantageous states is contrasted with that of peer-punishment. Chen *et al.*^20^ show that in the public goods game, the introduction of punishment has a positive effect on cooperation especially for large group size, while an intermediate group size is not best for cooperation. Sasaki *et al.*^21^ introduce the deposit that will be refunded as long as the committers faithfully cooperate in the donation game, and punish free riders and non-committers.

Here, contrary to those previous discussions, this study newly proposes the probabilistic peer-punishment based on the difference of payoff. To prevent the antisocial behaviour, the proposed peer-punishment enables a player to punish another uncooperative player having a connection with him/her and larger payoff than his/her payoff. The probability that a player punishes another uncooperative player is directly proportional to the difference between each payoff of punishing and punished players. Regarding the difference between the proposed peer-punishment and the ordinary peer-punishment^6^, the proposed peer-punishment has the following characteristics; the cost of a punishing player and the degree of punishment to another player are not constant, but dynamically change based on the payoff of a punishing player. This study is similar to the Fehr and Schmidt’s inequity aversion^22^, however, there is the difference between their inequity aversion^22^ and this study as follows; their model utilizes not the prisoner’s dilemma game, but the public goods game, and enables a player to punish not other players having connections with him/her, but all other players.

The author also describes the difference between the proposed peer-punishment and other probabilistic punishment as follows. Chen *et al.*^23^ consider probabilistic punishment as the simplest way of distributing the responsibility to sanction defectors. The probability of punishment is fixed among players, and does not change depending on the difference of payoff between each payoff of punishing and punished players. The following studies also discuss probabilistic punishment: class-specific probabilities of punishment that is based on the fixed number of classes^24^, the implicated punishment that has a working probability *p* (0 < *p* < 1), and includes the peer-punishment on defectors with a probability *q* (0 < *q* < 1)^25^. However, those probabilities are fixed, and also does not change. Szolnoki and Perc^26^ consider the conditional punishment that does not depend on the difference of payoff between each payoff of punishing and punished players, but is proportional to the number of other conditional and unconditional punishers within the group.

This study refines Ohdaira’s previous work^27^ by introducing the precise definition of the probability that a player punishes another uncooperative player, and shows that the introduction of the proposed peer-punishment effectively increases both the number of cooperative players and the average payoff of all players in various types of topology of connections. In addition, referring to the previous discussions regarding costly punishment mentioned above, the author describes new knowledge from this study.

## Model

This study is based on Nowak and May’s spatial prisoner’s dilemma game introducing connections^28^. Every player has each strategy of two types, defection (=defector) and cooperation (=cooperator), matches the other players having connections with him/her, and then acquires the cumulative payoff from all matches. When *N* is the number of all players, players of a match are *i* and *j* (*i* ≠ *j*, 1 ≤ *i*, *j* ≤ *N*), *s*(*i*) and *s*(*j*) are their strategy, and *P*(*i*) and *P*(*j*) are their payoff, *P*(*i*) is expressed as the equation (2) utilizing the payoff matrix *A* of the equation (1). Note that *s*(*i*) and *s*(*j*) are either (1 0) (cooperator) or (0 1) (defector) of unit vectors. *O*(*i*) denotes the set of the other players having connections with player *i*.


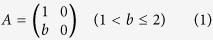



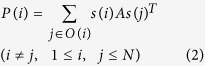


**Figure 1:**
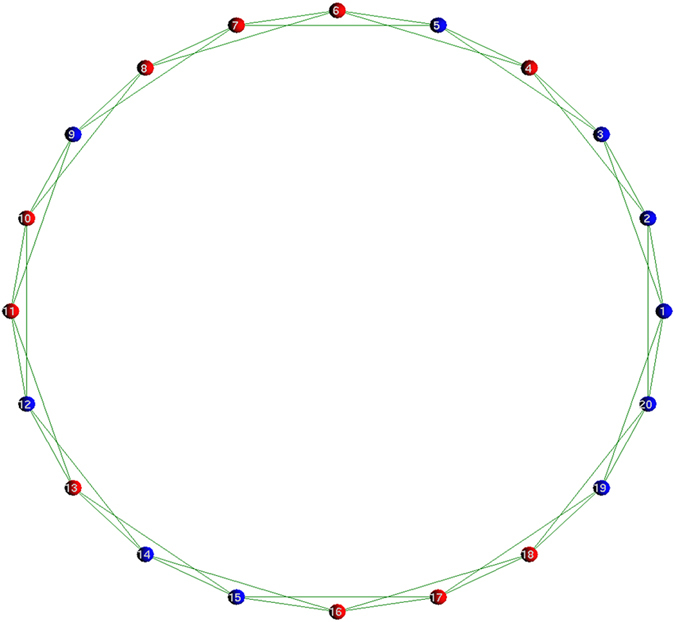
The sample initial state of the regular topology of connections of <*k*> = 4. The regular topology of connections means that the number of players having connections with player *i* (*k*(*i*)) is the same regarding all players. The spatial structure of connections is defined as the one dimensional lattice of periodic boundary conditions, and each vertex of the lattice exhibits each player. Note that this figure has only twenty players (*N* = 20) in order to make clear the spatial structure of connections. The ratio of the number of defectors (red) to the number of cooperators (blue) approximately equals one to one, and defectors and cooperators are randomly distributed in every simulation run. Each type of the topology of connections in each initial state does not change during every simulation run.

The spatial structure of connections of this study is based not on Nowak and May’s two dimensional lattice^28^, but on Santos and Pacheco’s model^29^. Therefore, it is defined as the one dimensional lattice of periodic boundary conditions (see Fig. 1). Each vertex of the lattice exhibits each player, and the number of players having connections with player *i* is *k*(*i*). The average of *k*(*i*), 
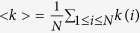
. Figure 1 exhibits the sample initial state when *k*(*i*) is the same regarding all players (i.e. the regular topology of connections, *k*(*i*) = <*k*> = 4, 1 ≤ *i* ≤ *N*). Note that Fig. 1 has only twenty players (*N* = 20) in order to make clear the spatial structure of connections. The ratio of the number of defectors to the number of cooperators approximately equals one to one, and defectors and cooperators are randomly distributed in every simulation run. That ratio also refers to Santos and Pacheco’s model^29^. The topology of connections is the following three types; the regular^30^, the (completely) random^30^, and the scale-free known as the Barabási-Albert model^31^. Each type of the topology of connections in each initial state does not change during every simulation run.

In the following, the author describes the implementation of the proposed peer-punishment. Dreber *et al.*^2^ introduce the punishment of a player to another player as strategy, while in this study, in order to prevent the antisocial behaviour, a player does not have the strategy of punishment, and punishes another uncooperative player of higher payoff than his/her payoff with the probability that is directly proportional to the difference between each payoff of punishing and punished players. When the payoff of player *i* (*P*(*i*)) is smaller than the payoff of player *j*∈*O*(*i*) (*P*(*j*)), i.e. *P*(*i*) < *P*(*j*) ≤ 2*P*(*i*), player *i* punishes player *j* with the probability *q*_*i*_(*j*) as the equation (3). When the inequality of *P*(*j*) > 2*P*(*i*) holds, *q*_*i*_(*j*) = 1.


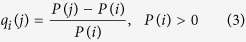


When player *i* punishes player *j*∈*O*(*i*) that satisfies the condition of *P*(*j*) > *P*(*i*) based on the proposed peer-punishment, *P*(*i*) and *P*(*j*) change into *P*(*i*)′ and *P*(*j*)′ as the following equation (4). Player *i* is not able to punish player *j* without the loss of payoff, and then has the same loss that is given to player *j*. Note that *r* is the coefficient of punishment, and player *i* is able to punish player *j* when the inequality of *P*(*i*)(1 − *rn*(*i*)) > 0 holds. The variable *n*(*i*) indicates the number of players *j*∈*O*(*i*) that satisfies the condition of *P*(*j*) > *P*(*i*).


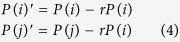


The decrease in the payoff due to punishing the opponent and being punished by the opponent is calculated independently, and eventually *P*(*i*)′ = 0 in the case of *P*(*i*)′ < 0. Therefore, *P*(*i*)′ cannot be negative. After the payoff of all players changes due to all punishing and punished activities, as the following equation (5), player *i* chooses the strategy of player *j*_max_∈*i*∪*O*(*i*) for his/her strategy of the matches of the next generation. When more than one player has the maximum payoff, player *i* randomly chooses the strategy of one of them. Each strategy of all players is synchronously updated.


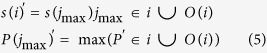


Parameter settings of this study are as follows based on Santos and Pacheco’s results of the scale-free topology of connections^29^; the average of *k*(*i*), <*k*> = 4, 8, and the number of all players, *N* = 1000. As noted before, in the initial state, the ratio of the number of defectors to the number of cooperators approximately equals one to one, and defectors and cooperators are randomly distributed in every simulation run. Each type of the topology of connections in each initial state does not change during every simulation run. The parameter in the payoff matrix *A*, *b* = 1.5 that is the intermediate value of the range of *b*. Regarding the coefficient of punishment, this study utilizes *r* = 0.15 because that value brings the greatest number of cooperators in each case of the regular topology of connections of <*k*> = 4, 8 within the range of 0 < *r* ≤ 0.3. The following results of both the frequencies of cooperators and defectors and the average payoff of all players until 300 generations are the average of 20 independent simulation runs, and basically have error bars of SD (standard deviation).

## Results

**Figure 2:**
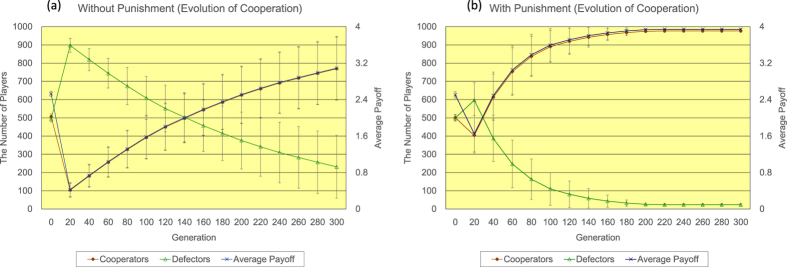
The time series results of the number of defectors (left vertical axis), the number of cooperators (left vertical axis), and the average payoff of all players (right vertical axis) regarding the regular topology of connections of <*k*> = 4 (**a**) without/(**b**) with the proposed peer-punishment. Note that error bars are SD (standard deviation).

**Figure 3:**
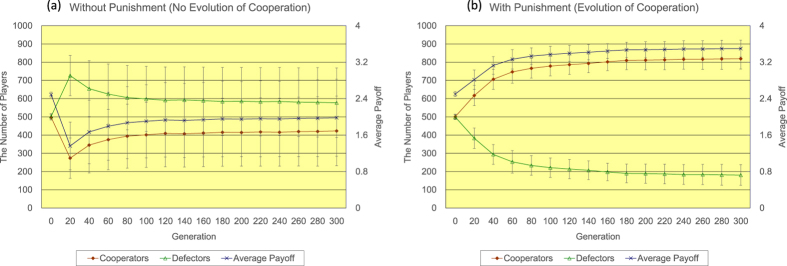
The time series results of the number of defectors (left vertical axis), the number of cooperators (left vertical axis), and the average payoff of all players (right vertical axis) regarding the random topology of connections of <*k*> = 4 (**a**) without/(**b**) with the proposed peer-punishment. Note that error bars are SD (standard deviation).

**Figure 4:**
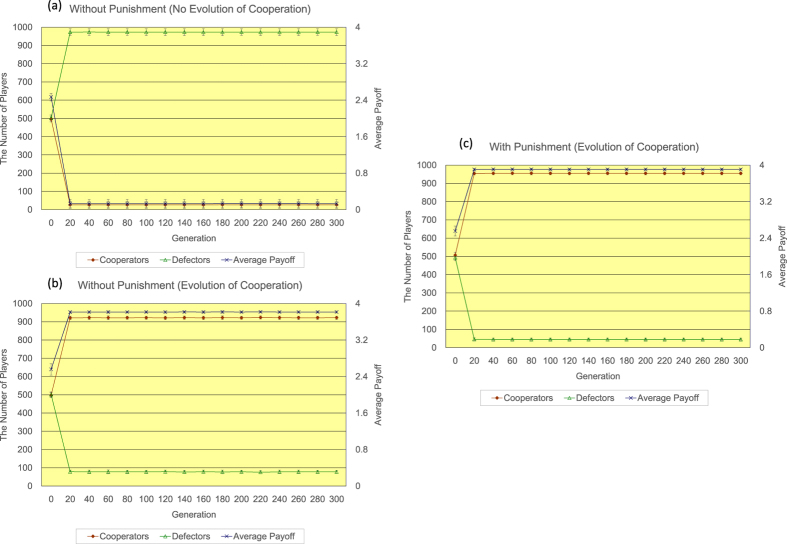
The time series results of the number of defectors (left vertical axis), the number of cooperators (left vertical axis), and the average payoff of all players (right vertical axis) regarding the scale-free topology of connections of <*k*> = 4. Regarding the case without the proposed peer-punishment, the panel (**a**) shows the case where the number of defectors increases (14/20 simulation runs), while the panel (**b**) shows the case where the number of cooperators increases (6/20 simulation runs). Regarding the case with the proposed peer-punishment (**c**), the number of cooperators increases in all simulation runs. Note that error bars are SD (standard deviation).

Firstly, the author shows the results of <*k*> = 4. Regarding the regular topology of connections (see Fig. 2), without the proposed peer-punishment (a), the number of defectors quickly increases, while the number of cooperators rapidly decreases until 20 generations. After the event, the number of defectors gradually decreases and the number of cooperators slowly increases until 300 generations. The number of cooperators eventually reaches approximately 769. On the other hand, with the proposed peer-punishment (b), almost all players come to cooperators in approximately 200 generations. The introduction of the proposed peer-punishment apparently enhances the increase in both the number of cooperators and the average payoff of all players. Regarding the random topology of connections (see Fig. 3), in the 300 generation, without the proposed peer-punishment (a), the number of cooperators is approximately 423, and the number of defectors is roughly 577, while with the proposed peer-punishment (b), the number of cooperators is approximately 819, and the number of defectors is roughly 181. Although we observe some decrease in the number of cooperators in comparison with the result of the regular topology of connections, the introduction of the proposed peer-punishment also increases both the number of cooperators and the average payoff of all players. Regarding the scale-free topology of connections (see Fig. 4), because the payoff of player *i* (=*P*(*i*)) is the resulting sum from several matches as well as Nowak and May’s model^28^, and is not normalized by the number of players having connections with player *i* (=*k*(*i*)) like Santos and Pacheco’s previous study^29^, both the number of cooperators and the average payoff of all players do not increase in the result without the proposed peer-punishment in 14/20 simulation runs (see panels of Fig. 4(a,b)). On the other hand, in the result with the proposed peer-punishment, almost all players come to cooperators in the 300 generation, and the average payoff of all players greatly increases in all simulation runs (see the panel of Fig. 4(c)).

**Figure 5:**
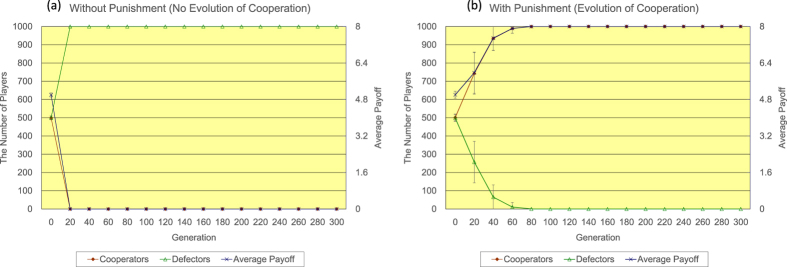
The time series results of the number of defectors (left vertical axis), the number of cooperators (left vertical axis), and the average payoff of all players (right vertical axis) regarding the regular topology of connections of <k> = 8. The panel (**a**) shows the result of the increase in the number of defectors without the proposed peer-punishment (17/20 simulation runs), while the panel (**b**) shows the result of the increase in the number of cooperators with the proposed peer-punishment (all simulation runs). Note that error bars are SD (standard deviation).

**Figure 6:**
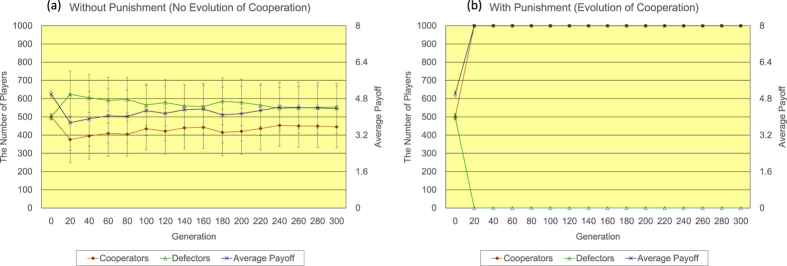
The time series results of the number of defectors (left vertical axis), the number of cooperators (left vertical axis), and the average payoff of all players (right vertical axis) regarding the random topology of connections of <*k*> = 8 (**a**) without/(**b**) with the proposed peer-punishment. Note that error bars are SD (standard deviation).

**Figure 7:**
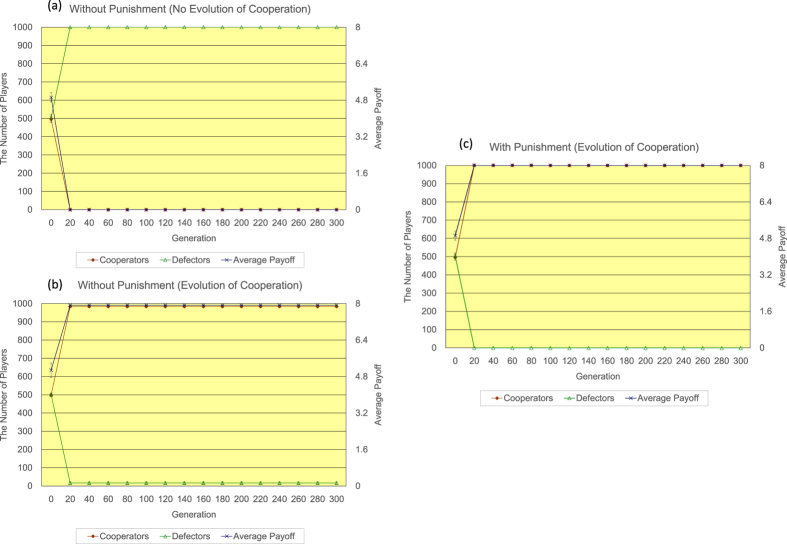
The time series results of the number of defectors (left vertical axis), the number of cooperators (left vertical axis), and the average payoff of all players (right vertical axis) regarding the scale-free topology of connections of <*k*> = 8. Regarding the case without the proposed peer-punishment, the panel (**a**) shows the case where the number of defectors increases (17/20 simulation runs), while the panel (**b**) shows the case where the number of cooperators increases (3/20 simulation runs). Regarding the case with the proposed peer-punishment (**c**), the number of cooperators increases in all simulation runs. Note that error bars are SD (standard deviation).

Secondly, the author shows the results of <*k*> = 8. Regarding the regular topology of connections (see Fig. 5), without the proposed peer-punishment (a), almost all players come to defectors in only 20 generations in 17/20 simulation runs, while with the proposed peer-punishment (b), almost all players come to cooperators in approximately 80 generations in all simulation runs. The introduction of the proposed peer-punishment apparently increases both the number of cooperators and the average payoff of all players. Regarding the random topology of connections (see Fig. 6), in the 300 generation, without the proposed peer-punishment (a), the number of cooperators is approximately 445, and the number of defectors is roughly 555, while with the proposed peer-punishment (b), almost all players come to cooperators in approximately 20 generations. The introduction of the proposed peer-punishment also contributes to the great increase in both the number of cooperators and the average payoff of all players as well as the result of the regular topology of connections of <*k*> = 8. Regarding the scale-free topology of connections (see Fig. 7), because *P*(*i*) is not normalized by *k*(*i*) as noted in the results of <*k*> = 4, without the proposed peer-punishment (see panels of Fig. 7(a,b), almost all players come to defectors in only 20 generations in 17/20 simulation runs. While with the proposed peer-punishment (see the panel of Fig. 7(c)), almost all players come to cooperators in approximately 20 generations, and the average payoff of all players greatly increases in all simulation runs. Those results show that the introduction of the proposed peer-punishment has an actual effect on the increase in both the number of cooperators and the average payoff of all players in every type of topology of connections of <*k*> = 8.

## Discussion

As noted in the introduction, Dreber *et al.*^2^ show that the introduction of peer-punishment decreases the average payoff of all players, and Rand and Nowak^5^ propose that peer-punishment is only a selfish tool to protect a player himself/herself because it facilitates the antisocial behaviour as a result of natural selection. In addition, Nowak^32^ shows that costly punishment is not the mechanism for the evolution of cooperation, but the supplement that enhances the degree of cooperation achieved by other mechanisms like indirect reciprocity, group selection, network reciprocity (connections) and so on. However, as shown in the results of this study, the proposed peer-punishment prevents the antisocial behaviour through its mechanism, strongly facilitates the evolution of cooperation in various types of topology of connections in addition to the regular topology of connections of <*k*> = 4 and the scale free topology of connections of each <*k*>, and contributes to the increase in the average payoff of all players. The proposed peer-punishment is myopic because it works between players having connections with each other. It diminishes the advantage of defectors in the spatial prisoner’s dilemma game, and is effective in decreasing the difference of payoff between players having connections with each other. Moreover, when a player punishes another player of higher payoff regardless of the difference between each payoff of a player and another player (i.e. constantly *q*_*i*_(*j*) = 1), the number of cooperators in the 300 generation greatly decreases approximately from 819 to 609 in the result of the random topology of connections of <*k*> = 4. This fact indicates that the introduction of the probability proposed in this study is universally effective in the evolution of cooperation in various types of topology of connections. As described in the results, without the proposed peer-punishment, the regular topology of connections of <*k*> = 4 has more opportunity for the evolution of cooperation than the random topology of the same <*k*>. This trend corresponds to Abramson and Kuperman’s result^33^ that when the parameter in the payoff matrix *A* (=*b*) equals 1.5 and <*k*> is 4, more randomness in the topology of connections induces the situation where defectors easily invade many more clusters of cooperators.

In the public goods game that is classified as the group interaction, the previous study^34^ reports that the avoidance of overpunishing is essential for the stable cooperation. Overpunishing means that the decrease of payoff of punished defectors is not constant, but variable depending on the number of punishers. In this study, defectors of high payoff are punished by many cooperators having connections with such defectors, however, the stable cooperation is achieved because the coefficient of punishment *r* is small. Regarding *r*, it is necessary to introduce the case where *r* is different between punishing and punished players in the future because defectors of high payoff possibly have some kind of power. In addition, it is also required to investigate whether the value of *r* = 0.15 is effective in the evolution of cooperation except for the topology of connections presented in this study or not, and to examine other values of *b*. The author has already started to investigate the effect of changing *r* in broader range than that utilized in this study. Those results will be presented by another forthcoming paper.

Iwasa and Lee^35^ also utilize the public goods game, and show that the graduated punishment is the most effective rule in the evolution of cooperation when the action of a player is incorrectly reported at a small probability, and the sensitivity of a player to the difference in the utility or payoff is not homogeneous. The graduated punishment means that the degree of punishment gradually changes based on the damage by selfish behaviour. The proposed peer-punishment is similar to the graduated punishment because the degree of punishment is directly proportional to the payoff of a player, and the probability of punishment of a player is also directly proportional to the difference between each payoff of punishing and punished players. The results of this study provide the interesting knowledge that the graduated-punishment-like proposed peer-punishment facilitates the evolution of cooperation in the spatial prisoner’s dilemma game that is classified as not the group, but the pairwise interaction. Helbing *et al.*^18^ investigate the evolution of cooperation in the spatial public goods game, and especially show that increasing the fine of punishment induces a rising of the level of cooperation, and larger punishment fines do not have any positive effects. Jiang *et al.*^36^ also describe that severe punishment is not necessarily more effective, and if cooperation is likely, mild punishment is not less effective, and leads to higher average payoffs. Those findings also conform to the results of this study well.

This study shows recent either negative^1^,^2^,^3^,^4^,^5^ or positive^6^,^7^,^8^,^9^,^10^,^11^,^12^,^13^,^14^,^15^ discussions regarding the effectiveness of costly punishment in the evolution of cooperation in non-kin relations. Contrary to those previous discussions, the author has newly proposed the probabilistic peer-punishment based on the difference of payoff. By introducing the proposed peer-punishment, antisocial behaviour is prevented through its mechanism, and also the flaw of costly punishment that the negative discussion^2^ designates is solved because both the number of cooperative players and the average payoff of all players effectively increase in various types of topology of connections. In the future, the author plans to advance this study following to the direction mentioned in the discussion.

## Methods

As described in the Model, the spatial structure of connections of this study is defined as the one dimensional lattice of periodic boundary conditions. Firstly, we construct the regular one dimensional lattice of periodic boundary conditions. “Regular” means that the number of players having connections with player *i*, *k*(*i*) is the same regarding all players. Secondly, by rewiring every connection of player *i* with the uniform probability *p* = 1, we build the random one dimensional lattice of periodic boundary conditions. As neither the deletion nor the duplication of every connection occurs, the average of *k*(*i*), <*k*> does not change after the rewiring. These procedures of the construction of the regular and the random lattice follow the method by Watts and Strogatz^30^. Thirdly, we set up the lattice of the scale-free topology following the method by Barabási and Albert^31^, i.e. starting with *m*_*0*_ = <*k*> + 1 players of a complete graph, at every time step we add a new player with *m* = <*k*>/2 edges that link the new player to *m* different players already present in the lattice until the total number of players (*N*) reaches 1000. A new player *i* will be connected to player *u* depending on *k*(*u*), so that *p*(*k*(*u*)) = *k*(*u*)/Σ*v k*(*v*) (*v*: the number of players already present in the lattice). Therefore, players of small *i* (≤<*k*> + 1) turn into high degree players. Each vertex of the lattice is initially occupied by either a defector or a cooperator at the same probability (1/2). Therefore, in the initial state, the ratio of the number of defectors to the number of cooperators “approximately” equals one to one. There is the case where the number of cooperators is a little larger than that of defectors, and vice versa. In the Supplementary Information, the author provides (1) the reason why cooperation evolves when introducing the proposed sanction utilizing the simple model (Figs. S1(a), S1(b), S1(c), and S1(d)); (2) the result of the scale-free topology of connections of <k>=4 and N=10000 that is enough to study effects on the scale-free topology of connections (Fig. S2); and (3) the result of the simulation on the random topology of connections of <k>=4 and N=1000 until 3000 generations that shows the stability of all results presented in the paper (Fig. S3).

## Additional Information

**How to cite this article**: Ohdaira, T. Evolution of cooperation by the introduction of the probabilistic peer-punishment based on the difference of payoff. *Sci. Rep.* **6**, 25413; doi: 10.1038/srep25413 (2016).

## Supplementary Material

Supplementary Information
