# Supplementary material for: Evolution of cooperation by the introduction of the probabilistic peer-punishment based on the difference of payoff
Source: Sci Rep. 2016 May 5;6:25413. doi: 10.1038/srep25413 (PMC4857114; doi:10.1038/srep25413)
Supplement: Supplementary Information [file srep25413-s1.pdf]

Supplementary Information: Evolution of cooperation by the introduction of the probabilistic peer-punishment based on the difference of payoff

Tetsushi Ohdaira[†]

[†]*Institute of Information and Media, Aoyama Gakuin University, 4-4-25 Shibuya, Shibuya-ku, Tokyo 150-8366 Japan*

In the Supplementary Information, firstly, the author provides the description that explains the reason why cooperation evolves when introducing the proposed peer-punishment utilizing the simple model (Figures S1(a), S1(b), S1(c), and S1(d)). Secondly, the author shows the result of the scale-free topology of connections of $<k>=4$ and $N=10000$ that is enough to study effects on the scale-free topology of connections (Figure S2). Thirdly, the author presents the result of the simulation on the random topology of connections of $<k>=4$ and $N=1000$ until 3000 generations that shows the stability of all results presented in the manuscript (Figure S3).

The reason why cooperation evolves when introducing the proposed sanction

We consider the simple initial state of $<k>=4$ and $N=20$ that consists of only a cluster of 4 cooperators and other 16 defectors (Figure S1(a)). In the case without the proposed peer-punishment, a cluster of 4 cooperators will spread in the lattice with the probability of 2/9. This probability is the product of the following 3 values of probability; each

defector (no.5 and 20) that is the nearest to the boundary of a cluster of 4 cooperators will respectively turn into a cooperator with the probability of 1/2, the boundary of a cluster of 4 cooperators (no.1 and 4) will respectively stay as a cooperator with the probability of 2/3, and the middle of a cluster of 4 cooperators (no.2 and 3) will respectively remain as a cooperator with the same probability. On the other hand, in the case with the proposed peer-punishment, each defector (no.5 and 20) that is the nearest to the boundary of a cluster of 4 cooperators will be punished by each neighbour defector (no.6 and 19) and cooperator (no.1 and 4). Therefore, each defector of no.5 and 20 will have smaller payoff than the payoff of their connected cooperators in a cluster of 4 cooperators (no.2 and 3), and always turn into a cooperator. Thus, the introduction of the proposed peer-punishment effectively enhances the prevalence of a cluster of 4 cooperators.

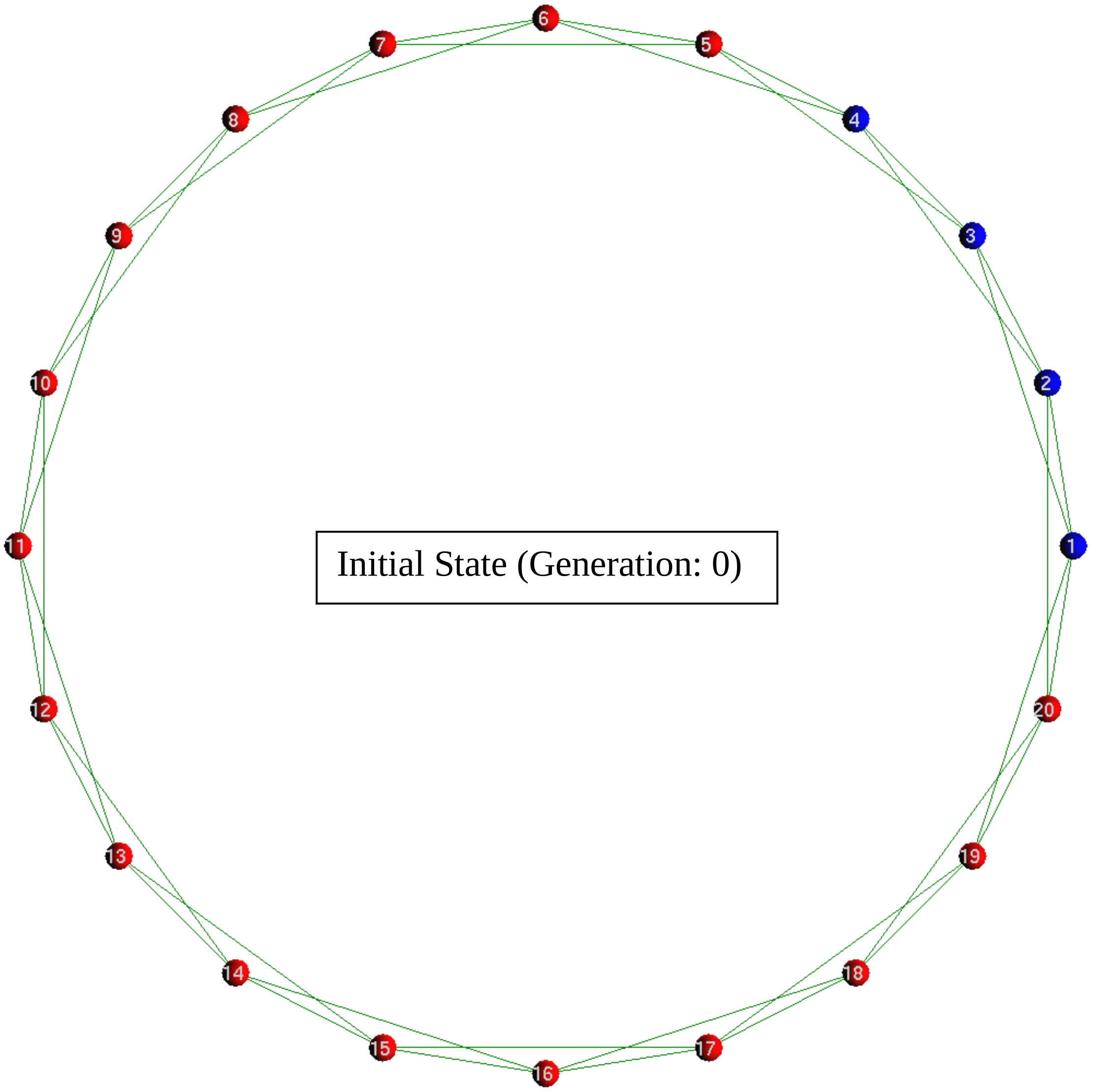

Figure S1(a). The evolution of cooperation until 3 generations in the case with the proposed peer-punishment when <k>=4, N=20, and only a cluster of 4 cooperators and other 16 defectors exist in the initial state. This figure shows the initial state of generation 0.

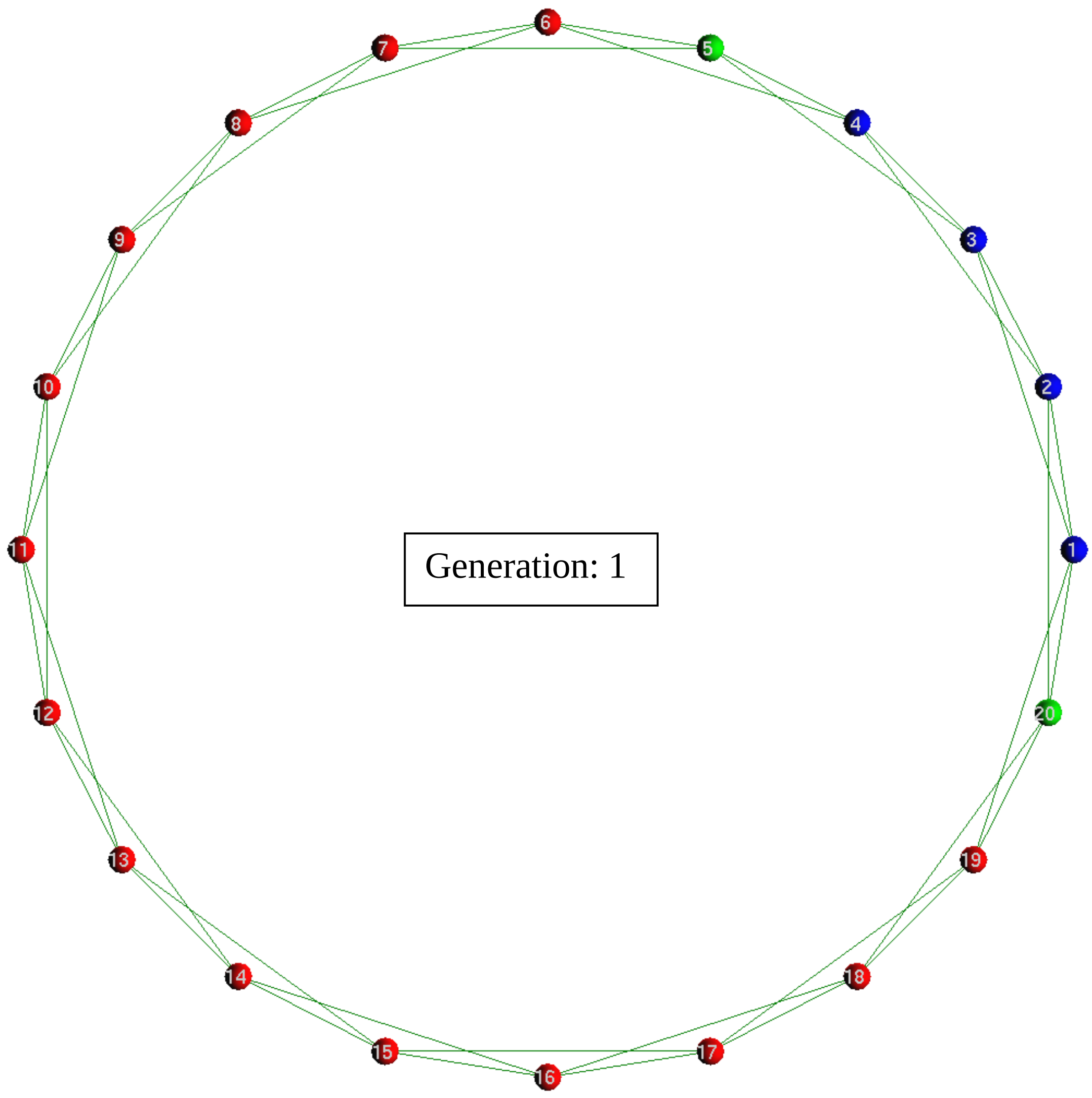

Figure S1(b). The evolution of cooperation until 3 generations in the case with the proposed peer-punishment when <*k*>=4, *N*=20, and only a cluster of 4 cooperators and other 16 defectors exist in the initial state. This figure shows the state of generation 1.

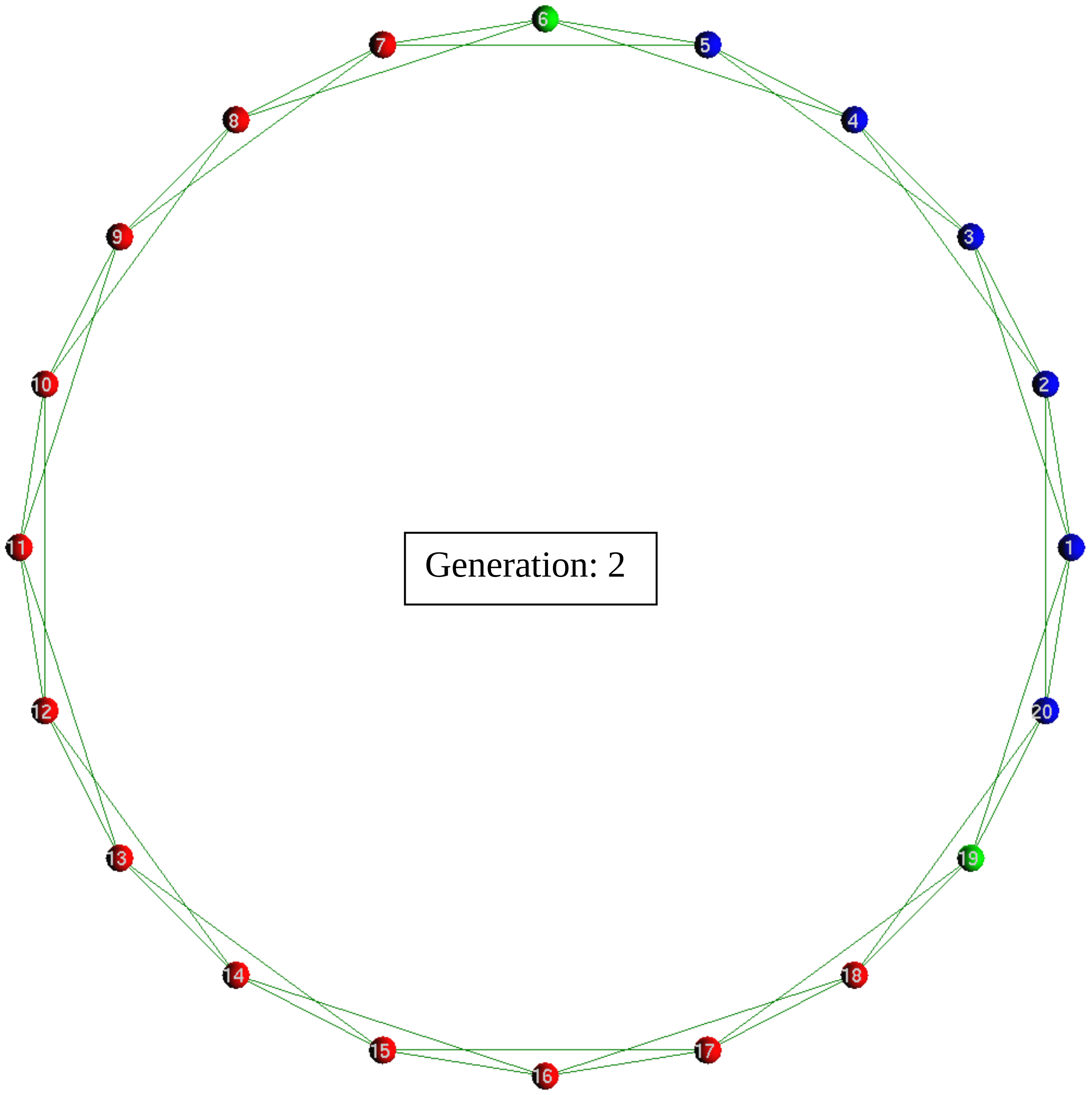

Figure S1(c). The evolution of cooperation until 3 generations in the case with the proposed peer-punishment when <*k*>=4, *N*=20, and only a cluster of 4 cooperators and other 16 defectors exist in the initial state. This figure shows the state of generation 2.

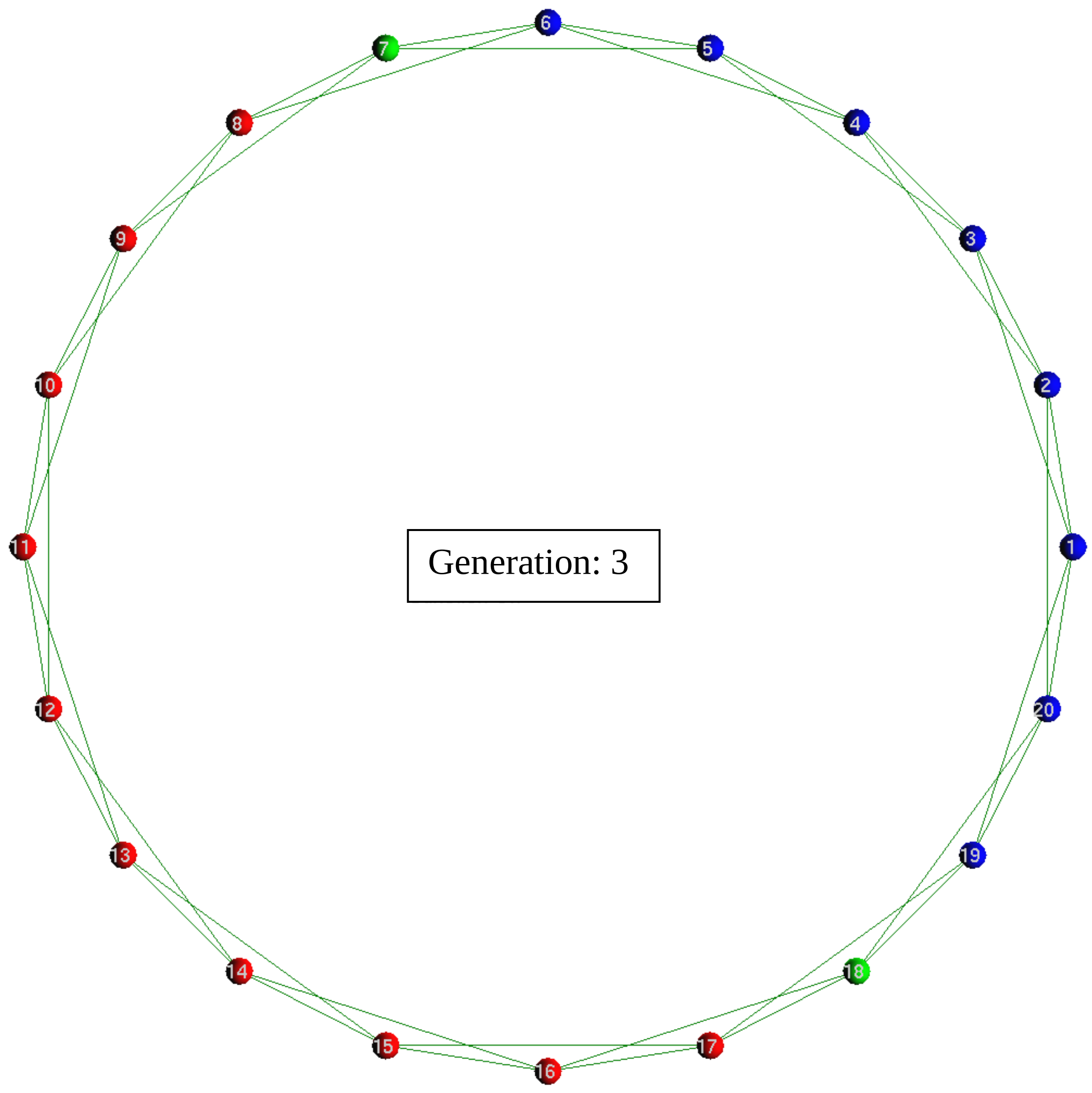

Figure S1(d). The evolution of cooperation until 3 generations in the case with the proposed peer-punishment when <*k*>=4, *N*=20, and only a cluster of 4 cooperators and other 16 defectors exist in the initial state. This figure shows the state of generation 3.

The result of the scale-free topology of connections of <*k*>=4 and *N*=10000

The author shows the result of the scale-free topology of connections of <*k*>=4 and *N*=10000 in the case with the proposed peer-punishment (see Figure S2). Almost all players come to cooperators in the 300 generation. This result indicates that the effect

on the evolution of cooperation by the proposed peer-punishment remains valid for significantly larger system size where scale-free character can reliably observed. The author believes that this system size of *N*=10000 is reasonable when discussing power-law relationships of the scale-free topology of connections (see Faloutsos et al., 1999).

Faloutsos, M., Faloutsos, P. & Faloutsos, C. On power-law relationships of the Internet topology. Proceedings of the conference on Applications, technologies, architectures, and protocols for computer communication (SIGCOMM ’99), pp.251-262 (1999).

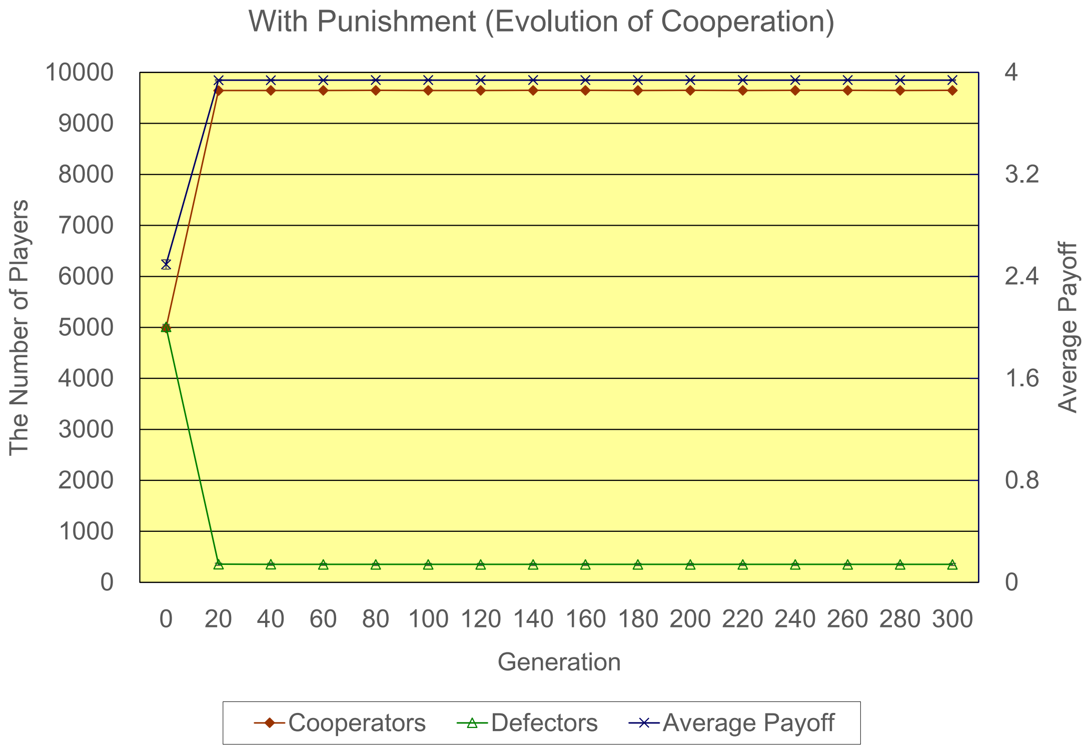

Figure S2. The time series results of the number of defectors (left vertical axis), the number of cooperators (left vertical axis), and the average payoff of all players (right vertical axis) regarding the scale-free topology of connections of <*k*>=4 and *N*=10000

with the proposed peer-punishment. The number of cooperators increases in all simulation runs. Note that error bars are SD (standard deviation).

The result of the simulation on the random topology of connections of <*k*>=4 and *N*=1000 until 3000 generations

Regarding the stability of the system, the author presents the result of the simulation on the random topology of connections of <*k*>=4 and *N*=1000 until 3000 generations (see Figure S3). This additional investigation indicates that all results presented in the manuscript are fully stable within 300 generations.

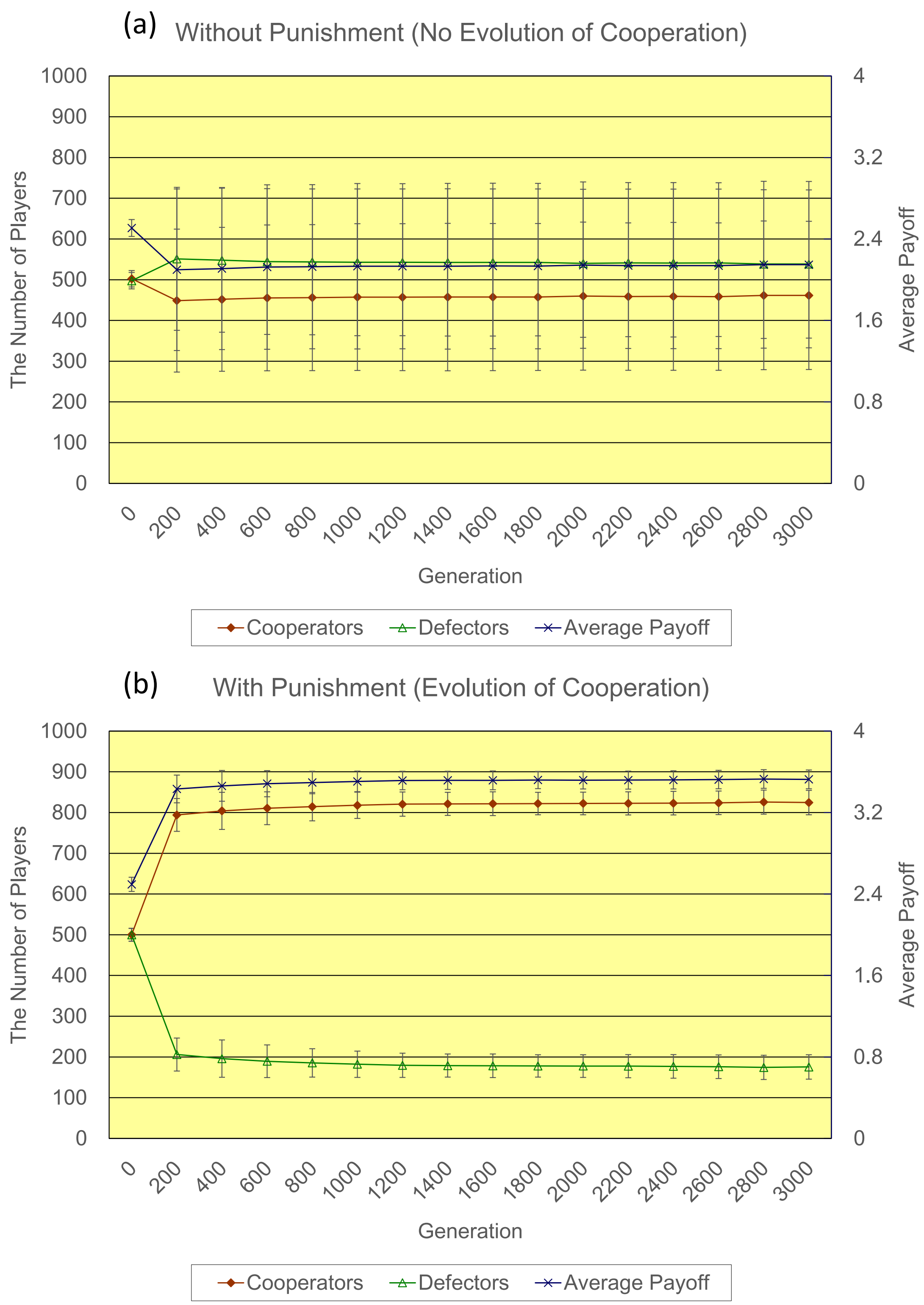

Figure S3. The time series results of the number of defectors (left vertical axis), the number of cooperators (left vertical axis), and the average payoff of all players (right

vertical axis) regarding the random topology of connections of $\langle k \rangle=4$ and $N=1000$ (a) without / (b) with the proposed peer-punishment until 3000 generations. Note that error bars are SD (standard deviation).
